# Unresectable Mesenteric Mass Causing Small Bowel Obstruction

**DOI:** 10.7759/cureus.29871

**Published:** 2022-10-03

**Authors:** Mary Zahnle, Sean M McCormack, Frederick Tiesenga, Juaquito Jorge

**Affiliations:** 1 General Surgery, Saint James School of Medicine, Park Ridge, USA; 2 School of Medicine, Saint James School of Medicine, Park Ridge, USA; 3 General Surgery, West Suburban Medical Center, Oak Park, USA; 4 General and Bariatric Surgery, West Suburban Hospital, Oak Park, USA

**Keywords:** net, abdominal pain, neuroendocrine tumor (net), unresectable, neuroendocrine tumor, sbo, small bowel obstruction

## Abstract

Small bowel obstruction (SBO) has a variety of etiologies, including but not limited to adhesions, malignancy, hernia, and inflammatory bowel diseases. Treatment for SBO may be nonoperative or operative, depending on the underlying condition and clinical symptoms. Clinical judgment and radiological findings cooperate in formulating an appropriate assessment and treatment plan. Mass effect due to malignancy is an indication for surgical intervention, as tumor resection is a mainstay of treatment. However, patient safety and chances of vascular compromise must be considered when determining if the tumor is resectable. Reported is a case of a 65-year-old female with severe abdominal pain, nausea, vomiting, and obstipation due to a malignant neuroendocrine tumor within the mesentery adjacent to the aortic bifurcation. Management included surgical intervention to alleviate bowel obstruction and biopsy of the tumor.

## Introduction

Small bowel obstruction (SBO) is a common condition and can be a surgical emergency. Clinical signs and radiological imaging are often used to diagnose SBO. When clinical signs and radiological imaging suggest SBO, this warrants moving SBO to the top of the differential diagnosis list, especially when the two are aligned. Similar to ours, a patient will present with acute abdomen features of nausea, vomiting, abdominal pain and distension, areas of hardness on and tenderness to palpation, and obstipation [1]. Abdominal computed tomography (CT) scans can aid in accurately diagnosing SBO, imaging that shows an abnormal gas pattern and transition point is highly suggestive of mechanical obstruction [1]. Patients diagnosed with acute SBO should be admitted to the hospital and receive a surgical consult. Immediate surgical exploration is indicated when clinical signs and symptoms suggest bowel compromise (i.e., perforation, necrosis, or ischemia). However, clinical signs are nonspecific and should not be used in insolation. Patients can safely undergo initial nonoperative management when surgical exploration is not immediately indicated. Nonoperative management initially includes IV fluid resuscitation, diet modification, and gastrointestinal decompression [2]. 

Nonoperative management resolves SBO in many patients, but success depends upon the etiology of the mechanical obstruction. The most common etiologies cited for SBO include adhesions due to prior abdominal surgery (70%), malignancy (10-20%), hernia (10%), and inflammatory bowel diseases (5%) [1]. The most commonly associated malignancies cited are ovarian, colonic, gastric, pancreatic, bladder, and endometrial [3]. Our patient presented with an unusual extra luminal and, in fact, intra-mesenteric malignant cause: a neuroendocrine tumor (NET). The annual incidence of NET tumors of clinical significance is 2.5-5 per 100,000 [4]. Traditionally classified by anatomic origins, NETs most commonly arise in the intestine, pancreas, and lung [5]. In this patient, the origin is unclear. NET is a relative indication for surgery as resection of the primary tumor improves patient outcomes. Interestingly, our patient's tumor proved to be within the mesentery just along the aortic bifurcation, causing a mass effect and fibrosis impeding the patency of her small bowel lumen. 

From a surgical perspective, SBO management includes predicting who will succeed and who will fail the nonoperative management of SBO. Operating on the latter early improves patient outcomes. However, accurately identifying the patients who are destined for surgery can be difficult. Our patient's radiological findings, which showed a NET, suggested surgical intervention is the treatment modality of choice [6]. Treatment included surgical removal of strictures caused by the malignant mass. Due to the tumor position directly bordering the aortic bifurcation, the mass cannot prudently be resected. Because NET may be responsive to specific chemotherapy [7], a biopsy of the mass was harvested to inform the subsequent treatment.

## Case presentation

A 65-year-old female presented to the emergency department due to abdominal pain for three days. The abdominal pain was associated with nausea and vomiting, dizziness, and obstipation for two days. The patient denied fever, chills, chest pain, shortness of breath, bowel changes outside of one-week duration, melena, or hematochezia. The patient endorsed a history of a cesarean section and a past medical history of hypertension, colon polyps, diverticulitis, and breast cancer over 20 years ago. She took no home medications and reported tobacco and tetrahydrocannabinol (THC) use.

Upon arrival, the patient was afebrile but uncomfortable. Her vital signs were stable and within normal limits, except for the previously diagnosed hypertension, which is unmedicated, with an oral temperature of 36.8°C, a heart rate of 90 BPM, a blood pressure of 175/95 mmHg, a respiratory rate of 18 BPM, and an oxygen saturation (SpO2) of 99% on room air. Upon examination, the abdomen was hard to the touch in the upper left quadrant while the patient was supine. The distended abdomen exhibited tenderness to deep palpation. Laboratory studies of blood and urine taken on the day of admission showed normal white blood cell count (8700/mcL), hemoglobin (14.0 g/dL), and hematocrit (42.8 g/dL) with elevated red blood cell count (5.75 x 10^6/mcl), blood glucose (114 mg/dL), calcium (10.6 mg/dL), and creatinine (1.36 mg/dL). Urine analysis revealed abnormalities of bacteria (2+), hyaline casts (5-10), ketones (trace), red blood cells (3-5), and squamous epithelial cells (5-10).

**Figure 1:**
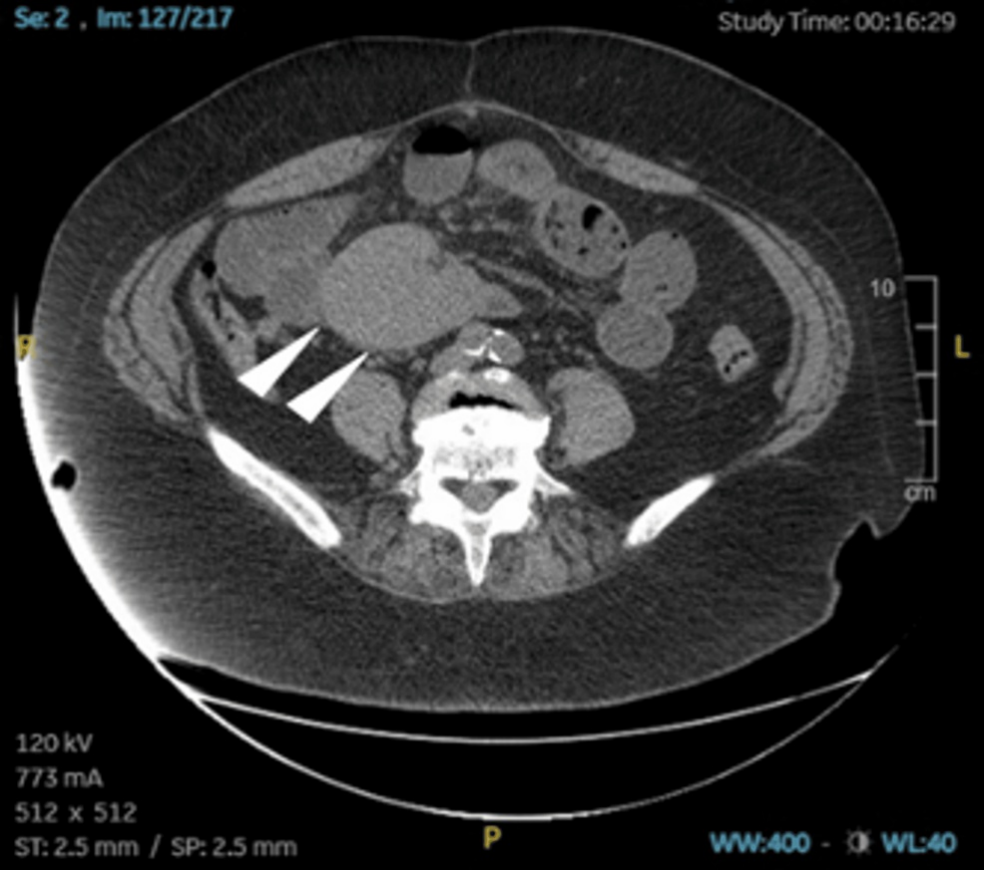
CT of the abdomen and pelvis without contrast, supine position, axial view displaying distended loops of small bowel, and adhesions near the NET (arrowheads) taken at initial ER presentation. NET: neuroendocrine tumor

**Figure 2:**
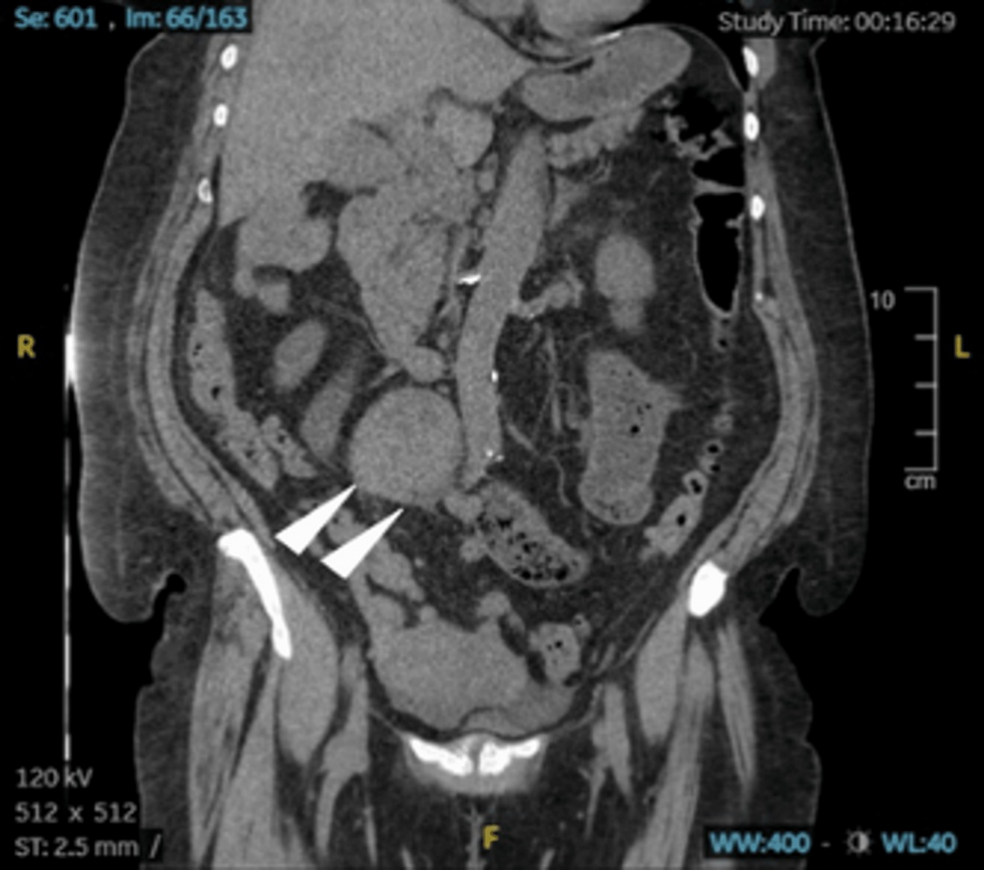
CT of the abdomen and pelvis without contrast, supine position, coronal view displaying distended loops of small bowel, and adhesions near the NET (arrowheads) taken at initial ER presentation. NET: neuroendocrine tumor

**Figure 3:**
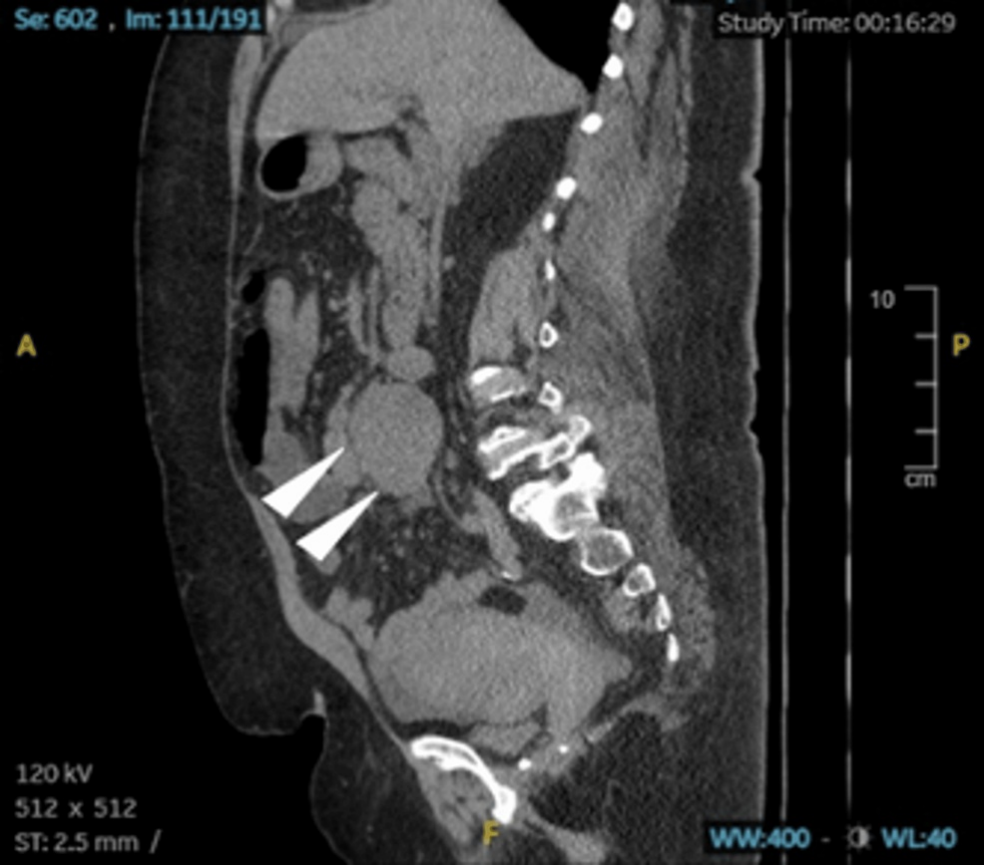
CT of the abdomen and pelvis without contrast, supine position, sagittal view displaying distended loops of small bowel, and adhesions near the NET (arrowheads) taken at initial ER presentation. NET: neuroendocrine tumor

Apart from her high creatinine level, unmedicated history of hypertension, and stage two hypertension reading in the emergency department, a CT of the abdomen and pelvis without contrast was impressive for abnormal bowel gas patterns consistent with small bowel obstruction. The transition point appeared to be associated with a large solid mass measuring 6.8 x 8.1 x 6.3 cm, apparently situated in the retroperitoneum at the root of the mesentery at the level of the aortic bifurcation. The mass appeared to have some associated tethering. The CT also showed subtle mass-like areas in the right liver, diverticulosis, and uterine fibroids (Figures 1, 2, 3). The patient was admitted to the hospital's family medicine services with a consult request sent to the surgical team. Initial treatment included nonoperative management, including IV fluid resuscitation and changing diet to nil per os (NPO; nothing by mouth). Due to emergent radiographic findings and immediate surgery team consultation, a nasogastric (NG) tube was not placed at this time. 

After the surgical team examination, concerns over the clinical and radiological findings were discussed with the patient. Open exploratory surgery with possible bowel resection, possible mesenteric mass excision, possible small bowel bypass, and possible proximal diverting ileostomy was recommended. In the case of the mesenteric mass being non-excisable, biopsy harvesting becomes necessary.

During open abdominal surgery, some omental adhesions to the anterior abdominal wall were discovered and taken down with electrocautery while progressing into the peritoneal cavity. Once the abdomen was entered and the small bowel eviscerated, attention came to a fibrotic band across the length of the small intestine. After removing strictures at this point, a large 8.5 x 9cm mass was found within the mesentery below, just adjacent to the bifurcation of the aorta. The small bowel proximal to the point was dilated and distally decompressed. Then the small bowel was examined from the ligament of Treitz to the transition point and then distally to the ileocecal valve. No other intestinal abnormalities were noted. Numerous lesions could be felt in the right liver lobe. As previously discussed with the patient, the mesenteric mass was determined non-excisable due to its profoundly adjacent location to a primary vascular structure of the aortic bifurcation and thus biopsied. A small bowel bypass was also performed. After ensuring the anastomosis was patent, perfused, and tension free; the abdomen was irrigated, closed with sutures, staples, and a wound vac; and a surgical drain was placed; the patient was extubated and taken to recovery in stable condition. 

Pathological examination of the biopsies taken during surgery revealed positive immunohistochemical staining for synaptophysin and cytokeratin AE1/AE3. The staining pattern supported the designation of a neuroendocrine neoplasm G2 with Ki67 at 6% and a mitotic rate of 2-3/10 hpf. The origin of the tumor was unclear. Based on this designation, liver lesion biopsies for confirmation were unnecessary, per the oncology team's recommendation. The next day, a Portacath was placed with no irregularities to facilitate eventual chemotherapy. 

The proceeding postoperative management focuses on ensuring a functioning small bowel with the patient having her first successful bowel movement on postoperative day six. One day of nasogastric tube decompression, six days of NPO, chewing gum, and daily ambulation facilitated this bowel movement. Management now focuses on complete wound healing and reaching optimal nutrition in preparation for chemotherapy recommendations.

## Discussion

Intestinal adhesions predominantly cause SBO. Most estimates attribute 70-75% of all cases to adhesions [1,8]. SBO caused by malignancy ranks a distant second, with estimates ranging from 5% [9] to 10-20% [1]. Colorectal, gynecological cancers, and melanoma make up the most common primary malignancy causing SBO via metastasis [9]. Primary adenocarcinoma of the small bowel is rare [10]. Other commonly cited causes of SBO include hernia, inflammatory bowel disease, and radiation. Several unique etiologies have been reported in the literature ranging from jejunal enteroliths [11] to small bowel volvulus [12], to sunflower seeds [13], and warfarin poisoning [14]. Our patient's neuroendocrine neoplasm in the mesentery is a rare cause of SBO.

NETs are a heterogeneous group of neoplasms thought to originate from neuroendocrine cells and their precursors throughout the body. The majority of NETs arise in the respiratory or gastrointestinal tract. Of the gastrointestinal tract, the typical locations include the small intestine (38%), rectum (34%), large intestine (16%), and stomach (11%) [15]. The intramesenteric area is not broken out into a percentage category upon review of the literature. These tumors are characterized by assorted yet often indolent biologic behavior. When not clinically silent, NET's clinical presentation is classically marked by their ability to secrete peptides resulting in specific hormonal syndromes illustrated by the following: carcinoid neoplasms causing flushing, cough, and diarrhea by releasing vasoactive substances such as serotonin; pancreatic insulinomas causing episodic hypoglycemia, which may cause confusion, visual change, unusual behavior, palpitations, diaphoresis, and tremulousness; gastrinomas presenting with peptic ulcers resistant to proton-pump inhibitors; glucagonoma presenting with varied features of diabetes mellitus, necrolytic migratory erythema, weight loss, diarrhea, venous thrombosis, cheilitis, and neuropsychiatric symptoms; VIPomas causing hypokalemic hypochloremic watery diarrhea [16]. Another clinical presentation is based on symptoms related to the mass effect of the tumor. When arising within the mediastinum, case reports indicate clinical features, including cough, dyspnea, chest pain, and superior vena cava syndrome [17]. Our patient similarly experienced the mass effect of her tumor as SBO. 

While specific details of the patient's breast cancer diagnosis from 20 years ago are unknown, it is interesting to note that one possible but rare type of breast cancer is neuroendocrine breast carcinoma (NEBC). NEBC accounts for 0.27-0.5% of all NETs and only 1% of breast cancer [18]. The literature sporadically reports NEBC may be metastatic NET, specifically from a primary tumor that is otherwise clinically silent [18]. Although very unlikely, it is possible our patient's breast cancer was a metastasis from the primary NET now causing her SBO. 

Noticeably, no randomized studies define small bowel-associated NETs' optimal surgical treatment [19]. Therefore, clinicians must rely upon the results of retrospective studies and personal experience in treating these patients. The North American Neuroendocrine Tumor Society (NANETS) published consensus guidelines in 2017 to summarize recommendations and treatment strategies. This patient's surgical management was aligned with the NANETS recommendations on safety. The first surgical goal noted is complete oncologic resection of the primary tumor(s) and mesenteric adenopathy/fibrosis while optimizing safety, operative time, quality of life, and cost [19]. Resection is a mainstay of treatment, and failure to resect the primary tumor can adversely affect the prognosis [20]. However, the proximity of the aorta to the mesenteric mass presents a challenge to clinicians when deciding on resection and patient safety. Guidelines note that when considering diseased lymph nodes at the root of the mesentery, attempting resection is not advised if compromise of mesenteric vessels is likely. Hence, the risk of vascular compromise presented by the bulk of our patient's tumor and lymph nodes precluded its complete resection and necessitates biopsy harvesting only. An additional NANETS gold standard recommendation for NETs is palpation of the small bowel's entire length, which was accomplished in the operating room. Palpation of the entire small bowel is a critical step in identifying small NETs and multifocal disease. While additional small NETs were not found, it is reported in 25-44% of patients that primary tumors are multifocal [19].

## Conclusions

This report outlined a case of a 65-year-old female who had clinical and radiological findings suggestive of SBO due to the mass effect and adhesions associated with a mesenteric NET, warranting surgical intervention. Surgical intervention can relieve the SBO syndrome but does not always ensure mass resection as well. Resection is usually the mainstay of treatment of NETs; however, due to the anatomic boundaries and neighbors of the mass, resection is not always the best management choice as in this case. Based on the NANETS guidelines and optimizing patient safety, the mass was biopsied rather than resected. The risk of vascular compromise presents a challenge to clinicians when deciding on resection. Similar cases should proceed cautiously with operative interventions based on clinical features and the tumor's location.
